# Notes and Notices

**Published:** 1892-02

**Authors:** 


### NOTES AND NOTICES.

Dr. WALTER E. HARVEY has removed from 154 Green Street, to 10 Magazine Street, Cambridgeport, Boston, Mass.

Dr. JOHN H. CLARKE, of London, England, has removed from Harrington Road, to No. 30 Clarges Street, W., Piccadilly.

E. B. TREAT, PUBLISHER, NEW YORK, has in press for early publication the 1892 *International Medical Annual*, being the tenth yearly issue of this deservedly popular work.

Its corps of thirty-five editors are specialists in their respective departments, and have been carefully selected from the brightest and best American, English, and French authors.

It is the embodiment of what is worth preserving of the current medical journals of the world for the year, and will contain six thousand references to diseases and their remedies.

The service rendered the profession by this annual cannot be over-estimated, and it is an absolute necessity to every physician who would keep abreast with the continuous progress of practical medical knowledge.

This index of new remedies and dictionary of new treatment, epitomized in one ready reference volume at the low price of $2.75, make it a desirable investment for the busy practitioner, student, and chemist.

THE BOSTON UNIVERSITY SCHOOL OF MEDICINE.—The homœopathic hospital attached to this college has grown to such proportions as to need a new building. The old building contained forty beds. The new building will accommodate two hundred and fifty. An interesting and detailed account of this project may be found in the Boston *Herald*, of December 26th, 1891.

THE DREVET MANUFACTURING Co., who manufacture the Peroxide of Hydrogen advertised in this Journal, have moved their laboratory to No. 28 Prince Street, New York City.

THE GLOBE PATENT AGENCY, of Washington, D. C., has issued a neat little book *On Patents and Kindred Topics*, telling just how to procure patents, copyrights, and trade-marks. It may be obtained free of Collamer & Co., 615 F Street, Washington, D. C.

THE AMERICAN ANTI-VACCINATION LEAGUE has recently been started in the United States for the purpose, as its name implies, of abolishing vaccination. Its officers include the names of some well-known homœopathic physicians, as will appear from a perusal of the following list: President, E. W. Sawyer, M. D., of Kokomo, Indiana; First Vice-Presideet, T. Dwight Stow, M. D., Mexico, New York; Second Vice-President, Alice B. Campbell, M. D., Brooklyn, N. Y.; Third Vice-President, Alexander Wilder, M. D., Newark, N. J.; Director, H. Hitchcock, M. D., New York; Corresponding Secretary, E. C. Townsend; Treasurer, Prof. O. M. Curtis, B. A., C. E.; Attorney, Thos. M. Wyatt. The headquarters of the League are in the office of Dr. Hitchcock, 19 Broadway, New York. Any one opposed to compulsory vaccination may become a member of the League on the payment of one dollar in annual dues. All are solicited to become members.

A REFUGE OF LIES.—Dr. Wm. F. Waugh, of Philadelphia, aptly terms the hypodermic syringe the refuge of lies, because so may doctors carelessly and hurriedly resort to it to bring their patients relief, rather than seek and root out the cause of pain.—*The Doctor.*

DR. MEYER BARUCH, who was one of the oldest German homœopathic physicians in New York was found dead on a lounge at his office, No. 183 Lexington Avenue, on Saturday afternoon, December 19th. He came to New York forty five years ago, and since then had been in active practice. He was seventy-five years old. The cause of his death was heart failure, brought on by work that was too hard for a man of his age. He was active in many charities and was a familiar figure to hundreds of people on lower Lexington Avenue. His widow, two daughters, and two sons, Drs. Emanuel and Simon Baruch, survive him.

W. B. SANDERS, publisher, 913 Walnut Street, Philadelphia, announces the following new books:

*An American Text-Book of Surgery.* By Professors Keen, White, Burnett, Conner, Dennis, Park, Nancrede, Pilcher, Senn, Shepherd, Stimson, Thomson, and Warren. Forming one handsome royal octavo volume of about 1,200 pages (10x7 inches), profusely illustrated with wood-cuts in text, and chromo-lithographic plates. Many of them engraved from original photographs and drawings furnished by the authors. Price, Cloth, $7.00; Sheep, $8.00.

*An American Text-Book of the Theory and Practice of Medicine, according to American Teachers.* Edited by William Pepper, M. D., LL. D., Provost of the University of Pennsylvania. To be completed in two handsome royal octavo volumes of about 1,000 pages each, with illustrations to elucidate the text wherever necessary. Price, per volume, Cloth, $5.00; Sheep, $6.00; Half Russia, $7.00.

Death in the Communion Cup.—Reading, November 8th.—Dr. John Ege of this city, who is paying special attention to bacteriology, in an interview says there is great danger in the method of administering communion.

"Do you believe that disease is communicated in passing the cup from one to another?" was asked.

"Most assuredly; not only by the communion cup, but by any other drinking vessel used by more than one person, or any article used by people with contagious diseases, or by the pernicious habit of kissing on the lips."

"How are the diseases communicated?"

"By means of the saliva from the mouth, which is left on the edges of the cup."

"Have you any remedy to suggest?"

"Communicants should be provided with their own cups, and when called to the altar receive the wine from the clergyman. I examined one drop of saliva on a glass used by a consumptive in the last stages, and found nearly a million of living tubercle bacilli in the single drop."—*From the Philadelphia Public Ledger and Daily Transcript.*

Patents.—The attention of our readers is directed to the advertisement of Munn & Co., patent solicitors, on fourth page of cover. Their name is familiar to patentees throughout the country. In connection with the publication of the *Scientific American* for the past forty-five years, they have made the drawings and specifications for more than one hundred and twenty thousand inventions, and their facilities for obtaining patents were never better than now.

The Association of Military Surgeons of the National Guard of the United States will hold its second annual session at St. Louis, April 19th, 20th, and 21st, 1892. An interesting programme of addresses by prominent surgeons of the National Guard and the United States Army has been arranged, and a goodly number of scientific papers on military and accidental surgery will be read and discussed, and all matters pertaining to the health, usefulness, and welfare of the civilian soldiers will receive attention. It is anticipated that not less than five hundred surgeons and assistant surgeons of the National Guard of the United States will be in attendance, to all of whom the Committee of Arrangements extend a most cordial welcome.

The Yale Surgical Chair, illustrated on advertising page 6 of this journal, is the most remarkable invention of the kind we have ever seen. It is everything that is claimed for it in the advertisement. We know whereof we speak, as a sample chair graces our office, where it may be seen by any member of the profession who is interested.

